# Parenting behaviors that shape child compliance: A multilevel meta-analysis

**DOI:** 10.1371/journal.pone.0204929

**Published:** 2018-10-05

**Authors:** Patty Leijten, Frances Gardner, G. J. Melendez-Torres, Wendy Knerr, Geertjan Overbeek

**Affiliations:** 1 University of Oxford, Centre for Evidence-based Intervention, Oxford, United Kingdom; 2 University of Amsterdam, Research Institute of Child Development and Education & Research Priority Area YIELD, University of Amsterdam, NG Amsterdam, the Netherlands; 3 Warwick Medical School, Division of Health Sciences, Gibbet Hill Campus, Coventry, United Kingdom; 4 Cardiff University, DECIPHer, School of Social Sciences, Cardiff, United Kingdom; 5 University of Glasgow, Institute of Health & Wellbeing, Glasgow, United Kingdom; Temple University, UNITED STATES

## Abstract

**Background:**

What are the parenting behaviors that shape child compliance? Most research on parent-child interactions relies on correlational research or evaluations of “package deal” interventions that manipulate many aspects of parenting at the same time. Neither approach allows for identifying the specific parenting behaviors that shape child compliance. To overcome this, we systematically reviewed and meta-analyzed available evidence on the effects of experimentally manipulated, discrete parenting behaviors—a niche in parent-child interaction research that contributes unique information on the specific parenting behaviors that shape child behavior.

**Methods:**

We identified studies by systematically searching databases and through contacting experts. Nineteen studies (75 effect sizes) on four discrete parenting behaviors were included: praise, verbal reprimands, time-out, and ignore. In multilevel models, we tested for each parenting behavior whether it increased child compliance, including both observed and parent-reported measures of child compliance.

**Results:**

Providing “time-out” for noncompliance robustly increased both observed and parent-reported child compliance (*d*s = 0.84–1.72; 95% CI 0.30 to 2.54). The same holds for briefly ignoring the child after non-compliance (*d*s = 0.36–1.77; 95% CI 0.04 to 2.90). When observed and parent-reported outcomes were combined, but not when they were examined separately, verbal reprimands also increased child compliance (*d* = 0.72; 95% CI 0.26 to 1.19). Praise did not increase child compliance (*d*s = –0.27–1.19; 95% CI –2.04 to 1.59).

**Conclusion:**

Our findings suggest that of the discrete parenting behaviors that are experimentally studied in multiple trials, especially time-out and ignore, and to some extent verbal reprimands, shape child compliance.

## Introduction

Parents’ attempts to socialize young children lead to a dynamic interplay between parenting behaviors to control child behavior and varying incidences of children’s compliance and non-compliance. Many forms of non-compliance in children are developmentally appropriate. Certain levels of resistance to parental control reflect children’s developing autonomy [1,2], one of the key aspects of healthy child development and well-being [3]. If children’s non-compliance rates rise above a certain threshold, however, this can reflect emotional and behavioral regulation problems, or problematic parenting, and can in some cases lead to the development of conduct problems [4].

Child compliance refers to the degree to which children do what parents ask them to do and refrain from doing what parents ask them not to do. Children comply with parental requests for different reasons. One main distinction is between willing compliance and coerced compliance [1,5]. Willing compliance reflects internally motivated compliance (i.e., children comply because they want to); coerced compliance, or obedience, reflects externally motivated compliance (e.g., to avoid threats or punishment, or to receive rewards). The intention of children’s compliance can be hard to judge. Examining the effects of parenting behaviors that are expected to activate either children’s internal or external motivation to comply may increase our understanding of why children comply.

### Why we need focused experimental research on parent-child interactions

Decades of research show associations between parenting behavior and children’s conduct problems [6–8]. This research is of paramount importance for our understanding of the undeniable link between parenting behavior and child compliance. Much of this research, however, has methodological limitations for building an understanding of the precise parenting behaviors that shape child compliance.

First, much research is correlational and thus cannot easily distinguish between causes and effects of parenting and child behavior. This is especially problematic given that children may influence parenting behavior as much as vice versa [9,10]. Besides, most of this research relies on broad parenting constructs, such as warmth and behavioral control [8]. These constructs are based on multiple and sometimes meaningfully different parenting behaviors. Parental warmth, for example, is a well-known predictor of compliant child behavior, especially when combined with appropriate levels of support and behavioral control [7]. Measures of warmth tend to include both sensitivity to children’s needs and expressing positive verbal and nonverbal affect. The latter in turn includes both unconditional expression of affection (e.g., daily set quality time to play) and conditional expression of affection (e.g., praise for compliance). If more warmth is associated with more child compliance, it remains unclear which elements of warmth (e.g., unconditional or conditional expression of affection) actually drive this association. The same holds for behavioral control, another well-known predictor of child compliance [7]. Parents adopt meaningfully different strategies to address children’s misbehavior. One distinction, for example, is between drawing attention to the fact that the child misbehaved (e.g., by giving verbal reprimands) versus temporarily withdrawing attention when the child misbehaved (e.g., ignoring the child or placing the child in “time-out” to prevent reinforcement of misbehavior). Relying on correlations between child compliance and broad parenting constructs such as warmth or control therefore provides limited insight into the precise parenting behaviors that shape child compliance.

Second, where experimental research is available, it typically tests the effects of complex multicomponent parenting interventions on children’s conduct problems (e.g., *Parent Management Training—Oregon Model*, *Triple P—Positive Parenting Program*, *Incredible Years*, and *Parent–Child Interaction Therapy* [11–14]). Studies on the effects of these comprehensive interventions are essential for informing clinical practice about the strategies that are most effective for reducing problematic levels of children’s noncompliance. However, because of their package-deal focus on simultaneously changing many different parenting behaviors, evaluations of these parenting interventions do not allow for pinpointing the exact parenting behaviors that shape child compliance [15].

Focused experimental research is relatively rare in parent-child interaction research. It is, however, precisely this approach that is necessary to identify the specific parenting behaviors that shape child compliance [15]. Often as precursors to comprehensive parenting intervention evaluations, many of these studies were conducted in the 1960s to 1980s. Behavioral experiments tested the effects of discrete parenting behaviors such as praise [16] and time-out [17] on child compliance. Recent work is adding to the body of evidence showing that discrete parenting behaviors can shape child compliance [18].

### The present systematic review and multilevel meta-analysis

The aim of the present meta-analysis is to identify the discrete parenting behaviors that shape child compliance, by analyzing the effects of individually manipulated parenting behaviors on children’s compliance. This aim is pursued with a quantitative multilevel meta-analytic approach that includes a comprehensive search for studies that experimentally tested the effects of parenting behaviors on child compliance.

## Methods

### Data sources, study selection, and inclusion criteria

We included studies that experimentally manipulated discrete parenting behaviors and tested the effects of these behaviors on child compliance. We identified studies: 1) through keyword searches in three databases (CINAHL, Embase and PsycINFO), including *child*, *parent*, *compliance*, *randomization* and varying examples of parenting behaviors, including *reinforcement*, *praise*, *time-out* etc (S1 Table); 2) by searching Web of Science, Scopus and Google Scholar using author names from known relevant studies; and 3) by emailing all authors of included studies to ask whether they knew of any other relevant studies. We last updated this search on February 7^th^ 2018.

We included experimental studies in which (i) the effects of the manipulation of a single parenting behavior was tested on child compliance; (ii) children’s mean age was 2–9 years (maximum age 13 years); and (iii) allocation to experimental and control condition was random. No restrictions were placed on the nature of the parenting behavior. For example, physical punishment was includable, but none of the studies that tested the effects of spanking fit inclusion criteria. No restrictions were placed on the nature of the control conditions, other than that they did not actively target the parenting behavior manipulated in the experimental condition. No restrictions were placed on language of the publication. We excluded studies that targeted parental feelings or cognitions (e.g., feelings of self-efficacy) rather than parenting behaviors.

Because we wanted to make sure to include all relevant rigorous research designs, we did additional systematic searches of the literature for (i) disentangling trials with multiple intervention conditions that differed on the specific parenting behaviors that are manipulated in each of the intervention conditions, and (ii) single-subject and multiple baseline studies that manipulated parenting behaviors and tested temporally associated changes in child compliance (S2 Table).

**Fig 1:**
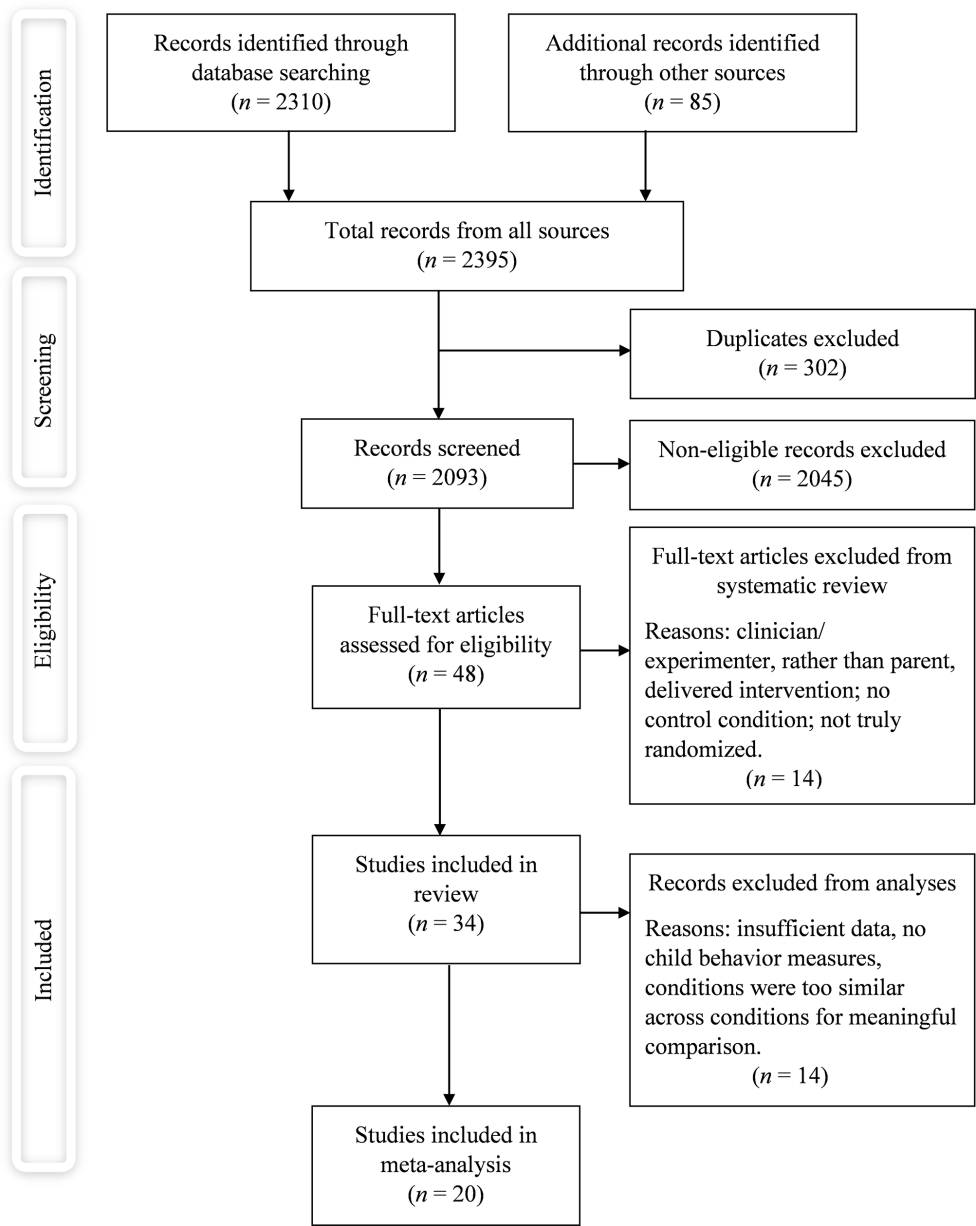
PRISMA flow diagram of included studies.

We first examined abstracts and, if needed, the full text, to produce a list of eligible studies (Fig 1). One author (WK) assessed abstracts and full texts; the final list of studies included in the review was assessed by two other authors (PL and FG; S3 Table). An overview of excluded studies and reasons for exclusion is included as Supporting Information (S4 Table).

### Data extraction

Studies were coded for (i) study characteristics (e.g., outcome measures included; risk of bias), (ii) sample characteristics (e.g., children’s age and whether children were typically developing, at-risk for non-compliant behavior, or referred for non-compliant behavior), and (iii) manipulated parenting behavior (e.g., “praise” or “time-out”). All studies were coded by two authors (PL and WK). Inter-coder agreement was excellent for both categorical characteristics (Cohen’s Kappa values >.95, e.g., lab versus home setting) and continuous characteristics (Intraclass correlations >.90, e.g., child age).

### Manipulated parenting behaviors

All manipulated parenting behaviors that were manipulated in multiple studies were included. This was the case for four parenting behaviors: (1) *Praise*, in which parents verbally express approval or admiration for the child’s compliance; (2) *verbal reprimand*, in which parents tell the child what s/he did wrong; (3) *Time-out*, in which parents take the child out of the situation in which noncompliance occurred, and place children in a separate part of the room, or another room, for a few minutes without social interaction; (4) *Ignore*, in which parents do not engage in any form of verbal or nonverbal interaction with the child for a few minutes. Please see S5 Table for our coding scheme.

Some studies further distinguished between different approaches to time-out (e.g., time-out procedures that included warnings versus procedures that did not include warnings) or different types of praise (e.g., labeled praise versus unlabeled praise). These subgroups of behaviors, however, were too scarce to be analyzed separately.

### Child compliance

Included outcome measures were observed and parent-reported child compliance (S6 Table). Assessments of other disruptive child behaviors, such as children’s hyperactivity of impulsivity, were excluded because these not necessarily reflect noncompliance. Measures of observed child compliance are generally preferred over parent-reported measures because they are less subjective, especially where raters are blinded to conditions [19]. Parent reports may be biased because parents themselves were the focus of the manipulation. Drawbacks of measures of observed child compliance are that they may be used in structured and lab-based settings that may be less natural, even if only because of the presence of a camera or observer, and cover the child’s behavior only in a particular setting and time [20]. In addition, observational tasks to assess compliance tend to differ meaningfully across studies in their level of structure (e.g., whether all parents provide children with the same set of instructions or spontaneous instructions are observed) and ecological validity (e.g., whether they are in the home setting with typical daily parenting instructions or in a lab setting with seemingly artificial instructions). The advantage of parent reports is that they can cover a broader range of child compliance in different settings (e.g., during morning routines and meal times) and at multiple times.

Because of the strengths and limitations of each approach, and because they can lead to meaningfully different outcomes [21], we decided to include both approaches and test them in separate models. In addition, because we know this is a small research field with a limited numbers of studies, we also tested parent-reported and observed outcomes together in one model, to gain statistical power. This approach to test effects both in separate models, and in a combined model, further allowed us to test whether findings are robust across type of outcome (i.e., parent-reported or observed).

### Effect size calculation

Effect sizes (Cohen’s *d*) reflected the standardized mean difference in child compliance between conditions with and without manipulation of the specific parenting behavior. Effect sizes were based on the raw means and standard deviations reported in the studies or obtained by contacting study authors. We preferred where possible to include post-test means and standard deviations that were ANCOVA-adjusted for baseline scores. If these were unavailable, we used unadjusted post-test means and standard deviations, or effect sizes estimated based on *t*-test and *F*-test statistics. When converting *F*-test statistics to Cohen’s *d*, we made conservative assumptions about the size of the difference. Positive effect sizes reflect that children in the experimental condition were more compliant than children in the control condition. A Cohen’s *d* of 0.20 reflects a small effect, 0.50 a moderate effect, and 0.80 a large effect [22].

### Multilevel approach

We used a multilevel meta-analysis approach, which has the benefit of preserving information from all relevant comparisons and outcome measures [23,24]. Each outcome of interest *j* is nested within a study *i*. In a multilevel meta-analysis, the effect size *d*_ij_ is derived from a sample, and has a sampling variance attached to it. We estimated models with three levels, where Level 1 was the participants’ outcome (and was thus implied, because our meta-analyses only has summary effect size estimates, not the individual participant data), Level 2 was the effect size associated with the comparison and outcome measure, and Level 3 was the study. That is to say, we nested effect sizes within studies. We placed random effects on both levels of the analysis.

We estimated three models for each parenting behavior: one on the effects of the parenting behavior on observed compliance, one on parent-reported compliance, and one on both observed and parent-reported compliance. We included the latter to increase the power of our analyses. We analyzed in each model all studies that compared the effects of the target parenting behavior with a control condition. We estimated all models in the R environment using the package–metafor–[25].

We assessed risk of bias of individual studies (as high, low or unclear) using the Cochrane Collaboration tool (Table 1). Specifically, we assess whether type of randomization procedure was clear, allocation was concealed, participants and outcome assessors were blind to conditions, incomplete data were addressed, drop-outs were analyzed, and the likelihood of selective outcome reporting. All studies reported random allocation, but especially older studies often failed to describe how sequences were generated and whether allocation was concealed). Because parents were actively instructed as part of the manipulation, participant blindness was not possible in any of the studies. Risk of bias was low for most studies on blinding of outcome assessors, addressing incomplete data, analyzing drop-outs, and selective outcome reporting. Across studies, the relatively small size of the field means the evidence-base we work with is relatively small, and not necessarily mature in terms of solid replication attempts.

**Table 1:** Risk of bias assessment of the included studies.

	Type of randomization procedure used is clear	Allocation concealment	Blinding of participants and personnel	Blinding of outcome assessors	Incomplete data addressed	Analyzed drop-outs	Selective outcome reporting
Acker (1996)	?	?	–	+	+	+	+
Adams (1992)	?	?	–	-	?	+	+
Bean (1981)	?	?	–	+	+	+	+
Bernhardt (1975)	?	?	–	?	+	+	+
Brock (2015)	+	+	–	+	+	+	+
Davies (1984)	?	?	–	+	+	+	+
Eisenstadt (1993)	+	?	–	+	+	+	?
Gardner (1976)	?	?	–	?	+	+	+
Kochanska (2013)	?	?	–	+	?	+	?
Leijten (2016a)	+	+	–	+	+	+	+
Leijten (2016b)	+	+	–	+	+	+	+
Odell (1982)	?	?	–	+	?	+	?
Reid (1999)	?	?	–	+	+	+	+
Reid (1994)	?	?	–	+	+	+	+
Roberts (1985)	?	?	–	?	?	?	?
Roberts (1988)	?	?	–	?	?	+	?
Roberts (1981)	?	?	–	+	?	?	?
Scarboro (1975)	?	?	–	?	?	+	?
Wahler (1997)	–	?	–	?	?	?	?

+ low risk of bias; − high risk of bias; ? unclear risk of bias.

## Results

### Included studies

We included 19 studies with a total of 75 effect sizes (Table 2). Children across studies ranged in age between one and twelve years, although most samples included children between the ages of 3 and 8 years. The majority of the studies (95%) tested the effects of manipulated parenting behavior in a single session and the majority of the studies (68%) were conducted in lab settings, as opposed to in the families’ homes. Independent observations of immediate compliance were included in 84% of the studies; parent-reported compliance was included in 26% of the studies. Less than half of the studies (47%) reported on the sample’s ethnicity. The majority of the families in the studies that did report ethnicity were white, with percentages of non-white families ranging 0 to 25. Almost all studies were conducted in the US; two studies were conducted in the Netherlands.

**Table 2:** Characteristics of included studies.

First author	Year	Parenting behavior	*N*	Child age range (*M*)	% boys	% not white	Sample	Setting	#sessions	Researcher allegiance	Observed outcome	Parent-reported outcome
Acker	1996	IG;VR	68	1.5–2.5 (2)	50	?	T	Lab	1	No	Compliance	–
Adams	1992	TO;VR	30	1–12 (5.72)	45	~ 0	AR	Lab	4	No	–	HRC
Bean	1981	TO	24	2–6 (3.6)	69	?	C	Lab	1	No	Compliance	–
Bernhardt	1975	PR	20	5–6 (5.5)	50	0	T	Lab	1	No	Compliance	–
Brock	2015	PR	186	1–3.5 (2.5)	52	25	AR	Home	1	Yes		ITSEA;ECI-4
Davies	1984	IG	80	3–7.5 (4.92)	54	?	T	Lab	1	No	Compliance	–
Eisenstadt	1993	PR; VR;IG;TO	24	2.5–7 (?)	92	12	C	Lab	7	Yes	Compliance	ECBI; CBCL-ext
Gardner	1976	TO	32	3.5–6.5 (?)	?	?	T	Lab	1	No	Compliance	–
Kochanska	2013	PR	102	2–3 (2.5)	52	25	T	Home	8	Yes	Compliance	–
Leijten	2016a	PR	161	4–8 (5.6)	45	12	T	Home	1	No	Compliance	–
Leijten	2016b	PR	132	3–10 (8.4)	71	9	AR	Home	1	No	–	ECBI; CBCL-aggr
O’Dell	1982	PR	100	2–10 (4.4)	34	33	T	Home	2	No	Compliance	–
Reid	1999	IG;PR	49	1.5–3.5 (?)	50	7	AR	Home	1	No	–	CBCL-ext
Reid	1994	VR	20	1.5–3.25 (2)	50	?	AR	Lab	1	No	Compliance	–
Roberts	1978	TO	27	3–7 (4.2)	?	?	AR	Lab	1	No	Compliance	–
Roberts	1981	TO	32	2–7 (3.7)	69	?	C	Lab	1	No	Compliance	–
Roberts	1985	PR	20	2–6 (3.5)	85	?	C	Lab	1	No	Compliance	–
Scarboro	1975	TO	24	4–6 (5.5)	38	?	T	Lab	3	No	Compliance	–
Wahler	1997	PR	36	? (7.45)	46	?	T	Lab	1	No	Compliance	–

VR = Verbal reprimand, TO = time-out, PR = praise, IG = ignore; Sample: T = typically developing sample; AR = at risk sample; C = clinically referred sample; Researcher allegiance = authors had a potential conflict of interest in the evaluated therapy; HRC = Home Report Card for aggressive child behavior, ITSEA = Infant-Toddler Social and Emotional Assessment, ECI-4 = Early Childhood Inventory; ECBI = Eyberg Child Behavior Inventory Intensity Scale, CBCL-ext = Child Behavior Checklist Externalizing Scale, CBCL-aggr = Child Behavior Checklist Aggression Scale.

Twenty-one percent of the studies included children clinically referred for conduct problems, 32% included children at risk for the development of conduct disorders (e.g., children with elevated levels of conduct problems), and 47% included typically developing children. Importantly, study characteristics (e.g., referred children versus typically developing children) did not appear to be confounded with the type of parenting behavior tested (e.g., time-out versus praise). The effects of all parenting behaviors were tested in multiple samples with different levels of conduct problems (Table 2).

Our additional searches for studies using rigorous designs other than focused experiments (i.e., disentangling trials, single subject and multiple baseline studies) did not lead to further eligible studies for inclusion in the meta-analysis. Of the disentangling trials (*k* = 11), ten studies did not meet inclusion criteria, in most cases because the differences between conditions was something other than teaching parents different parenting techniques. Please see the Supporting Information for excluded studies and reasons for exclusion. One study was already included as a focused experimental study [26]. Of the identified single-subject and multiple baseline studies (*k* = 4), only one study [27] provided the statistical details needed for meta-analysis. The three other studies did not provide these details [28–30]. Even if all single-subject studies had provided the statistical details needed for meta-analysis, the sparse number of subjects across all studies (*n* = 12), and the small number of crossover periods, precluded the robust use of meta-analysis methods for single-subject studies (e.g., multilevel models similar to individual participant data meta-analysis [31–33]).

### Parenting behaviors that shape child compliance

Two parenting behaviors significantly increased observed child compliance: providing time-out for noncompliance (*d* = 1.72, *p* < .001) and ignoring for noncompliance (*d* = 0.36, *p* < .001; Table 3). Providing praise for compliance or providing a verbal reprimand for noncompliance did not increase child compliance (*d*s range -0.27 to 0.74, *p*s > .09, respectively).

**Table 3:** Effects of parenting behaviors on increased observed and parent-reported child compliance.

Behavior	Outcome	*k* (*n*)	Cohen’s *d*	95% CI	Study level I^2^
Praise	Observed only	5 (9)	–0.27	–2.04, 1.50	74
	Parent-reported only	3 (8)	1.19	–0.81, 3.18	79
	*Combined*	7 (17)	0.20	–1.18, 1.59	79
Verbal reprimand	Observed only	2 (3)	0.74	–0.12, 1.60	0
	Parent-reported only	2 (4)	0.72	–0.05, 1.48	37
	*Combined*	3 (7)	0.72**	0.26, 1.19	0
Time-out	Observed only	6 (14)	1.72***	0.89, 2.54	65
	Parent-reported only	2 (4)	0.84**	0.30, 1.38	7
	*Combined*	7 (18)	1.57***	0.84, 2.29	65
Ignore	Observed only	3 (26)	0.36***	0.15, 0.57	0
	Parent-reported	2 (6)	1.77**	0.65, 2.90	27
	*Combined*	4 (32)	0.88*	0.04, 1.72	63

*k* = number of studies; *n* = number of effect sizes

*** *p* < .001

** *p* < .01

* *p* < .05

I^2^ reflects the degree to which heterogeneity in effectiveness between studies is greater than would be expected by sampling error alone. As a guideline [34], less than 30% is considered possibly unimportant heterogeneity, whereas more than 70% is considered substantial heterogeneity.

Consistent with our findings for observed child compliance, time-out (*d* = 0.84, *p* < .01) and ignore (*d* = 1.77, *p* < .01) increased parent-reported child compliance, whereas praise and verbal reprimand did not (*d*s were 1.19 and 0.72, *p*s > .07).

When we tested the effects of each parenting behavior on child compliance across the two different types of outcome measures (i.e., including measures of both observed and parent-reported compliance), we found that not only time-out (*d =* 1.57, *p* < .001) and ignore (*d* = 0.88; *p* < .05) increased child compliance, but that verbal reprimands did so too (*d* = 0.72, *p* < .01). Also across outcome measures, however, praise did not increase child compliance (*d* = 0.20, *p* = .777).

Were we to have had 10 or more studies in any one comparison, we would have used Egger’s test to examine small-study and publication bias. We chose not to use funnel plots as these would have been misleading with multiple effect sizes per study.

## Discussion

We examined the extent to which discrete parenting behaviors shape child compliance. We evaluated evidence from focused experimental research on parent-child interactions, where discrete parenting behaviors were manipulated to examine their effects on child compliance. This type of research is relatively rare in parent-child interaction research, but adds unique information on the precise parenting behaviors that shape child compliance.

Parenting behaviors that robustly increased child compliance across outcome measures (i.e., observed and parent-reported) were using a time-out procedure when children do not comply, and ignoring children for a few minutes when they do not comply. When both types of outcome measure were combined, but not when observed and parent-reported outcomes were examined separately, verbal reprimands also increased child compliance. Praise did not increase child compliance.

Patterson’s coercive process model [35] suggests that preventing reinforcement of noncompliance is the most effective way to increase child compliance. Placing children in time-out for noncompliance, or briefly ignoring them, are ways of preventing reinforcement. In a time-out procedure the child is isolated from social interaction and other reinforcers by being taken out of the situation where s/he was noncompliant and placed in another room, or another part of the room. In an ignore procedure the child stays in the situation where s/he was noncompliant, but does not get any attention from the parent. Time-out procedures might in some cases affect child compliance through other mechanisms than social isolation alone, such as removing the child from an enjoyable activity. Importantly, however, time-out and ignore share an important characteristic with each other that they do not share with other negative consequences such as natural consequences or taking away privileges: they briefly isolate the child from interaction with the parent. As such, they may activate children’s innate basic psychological need to belong [36], and therefore their motivation to reconnect with the parent.

### Why were some parenting behaviors more effective than others?

There is evidence to suggest that “bad is stronger than good” and that parenting behavior that is unpleasant for the child affects children stronger than parenting behavior that is pleasant for the child [37]. Our findings in part support this hypothesis, by suggesting that mainly disciplining behaviors (i.e., time-out and ignore, and in part verbal reprimand), as opposed to praise, improve immediate and short-term child compliance. This is in line with findings that the short-term effects of negative consequences on child compliance are fairly consistent, whereas the effects of praise and nurturance on child compliance are less consistent [38,39].

Heterogeneity was especially large between studies that tested the effects of praise on child compliance. Praise is controversial. On the one hand, research on the development, prevention, and treatment of conduct problems generally suggests that praise is part of a positive parenting style that protects against the development of conduct problems, and is effective in reducing conduct problems [40]. On the other hand, research on children’s motivation and prosocial behavior suggests that praise can undermine children’s intrinsic motivation [41–42]. Praise tends to be perceived as positive, but also as controlling, because praise is provided contingently upon specific behavior only [43]. Thus, praise can yield both positive and negative effects, depending on precise wording, to whom it is provided, and the context in which it is provided. The heterogeneity that we observed may well reflect these divergent patterns.

### Possible changes over time

Studies included in our meta-analysis focused exclusively on immediate and short-term effects of parenting behaviors, with studies varying from several minutes to multiple weeks in the time lag between manipulating and outcome. Some parenting behaviors may be slower to influence child compliance than other parenting behaviors, and some may not even intend to evoke immediate responses, but have longer-term goals such as strengthening the parent-child relationship. Similarly, some parenting behaviors that influence immediate compliance may lose their effects over time. Corporal punishment, for example, is related to immediate compliance but not to longer-term compliance, and is inversely related to children’s conduct problems [44,45]. The relative contributions of different parenting behaviors over time remain unclear and warrant further investigation.

### Possible additive or synergistic effects of parenting behaviors

We tested the effects of individual parenting behaviors on child compliance. Combined parenting behaviors sometimes have stronger effects on child behavior than individual parenting behaviors [46]. One of the most prominent hypotheses in this context is that teaching parents relationship building and nurturing skills increases the effects of negative consequences on child behavior, because negative consequences will then be more strongly associated with the loss of a valued positive reinforce [47]. Very few studies are set up to test such a two-stage model. Yet, our findings do suggest that the most powerful consequences might be ones in which children lose the valued positive reinforcements of parental acceptance and interaction. Future work is needed to identify whether improving parental relationship building and nurturing skills indeed increases the effects of disciplining behavior on child behavior.

### Is increasing child compliance a good thing?

Distinctions such as those between willing compliance and coerced compliance [1,5] illustrate the complexity of judging child compliance as either a desirable or an undesirable outcome, and a narrow focus on child compliance as a desirable outcome is an oversimplification of longer-term child well-being. Most notably perhaps, physical punishment can increase immediate child compliance, but has detrimental effects on child well-being and long-term conduct problems [44,45]. Some research suggests that time-out and ignore procedures also have negative side-effects for children. Social pain, the emotional reaction to being excluded from desired relationships, can hurt as much as physical pain [48]. In this study, we do not address whether for example time-out and ignore are either adequate or inadequate parenting behaviors, and whether child compliance caused by these procedures is either desirable or undesirable. Our study only shows that time-out and ignore promote immediate and short-term child compliance.

### Informing intervention strategies

Our findings provide insights into the parenting behaviors that seem most effective at increasing immediate and short-term child compliance. They cannot directly inform parenting interventions about the behaviors they should, or should not, teach parents to reduce problematic levels of non-compliance or conduct problems. As discussed, some parenting behaviors may need more time to influence child behavior. Our findings should be integrated with findings from complementary research strategies, such as meta-analysis of the associations between parenting intervention components and intervention effects [49,50] and longitudinal studies on bidirectional relations between various types of parenting behavior and child compliance [51,52], to understand the empirical merit of implementing discrete parenting behaviors as part of intervention strategies.

### Strengths and limitations

Our study is the first systematic examination of the discrete parenting behaviors that shape child compliance. This work fills a critical gap in our knowledge on child compliance that often relies on correlational designs and complex intervention evaluation research. Our study disentangles different aspects of broad parenting constructs such as behavioral control into discrete parenting behaviors such as verbal reprimands, ignore, and time-out. We conducted all analyses in three parallel models (observed and parent-reported child compliance, and both outcomes combined). Results were replicated across models for almost all findings.

However, our study is not without limitations. First, the quality of all meta-analyses depends on the characteristics of the primary studies—ours is no exception. We focused exclusively on immediate (observed) and short-term (parent-reported) child compliance, because none of the studies included measures of child behavior beyond several weeks—most included relatively immediate measures of child compliance only. Importantly, the aim of our study was to take a close-up shot of how parenting behavior shapes child compliance. The aim of our study was not to test long-term effects of parenting behavior on child outcomes. Second, and relatedly, the primary studies provide empirical support for the effects of parenting behaviors on child compliance, but not on the mechanisms that presumably underlay these effects. Third, the number of available studies was relatively small, despite drawing on 40 years of cumulative research and comprehensive attempts to locate different relevant bodies of evidence (i.e., disentangling trials, single-subject and multiple baseline studies). One of the consequences of the limited number of studies is that we did not have sufficient statistical power to test whether the effect of parenting behaviors depends on the extent to which another parenting behavior is used (i.e., interaction effects).

## Conclusion

We identified discrete parenting behaviors that causally affect child compliance. Based on the available evidence, we found that time-out and ignore procedures increased child compliance, robustly across observed and parent-reported outcomes. There was some evidence, though less robust, that verbal reprimand increased child compliance. Praise did not affect child compliance. More generally, more focused experimental research on parent-child interactions is needed to improve our understanding of the specific parenting behaviors that shape child compliance.

## Supporting information

S1 Checklist PRISMA checklist. (DOC)

S1 Table General search strategy. (DOCX)

S2 Table Additional search strategy for disentangling trials. (DOCX)

S3 Table References of included studies. (DOCX)

S4 Table Excluded studies and reasons for exclusion. (DOCX)

S5 Table Coding scheme for classifying parenting behaviors. (DOCX)

S6 Table Coding scheme for classifying outcome measures. (DOCX)
