# Supplementary material for: Parenting behaviors that shape child compliance: A multilevel meta-analysis
Source: PLoS One. 2018 Oct 5;13(10):e0204929. doi: 10.1371/journal.pone.0204929 (PMC6173420; doi:10.1371/journal.pone.0204929)
Supplement: S1 Table — (DOCX) [file pone.0204929.s002.docx]

**S1 Table. General Search Strategy.**

**Search terms for PsycINFO and Embase**

| Child | Cooperation | |
| --- | --- | --- |
| Child behavior | Cooperative Behavior | |
| Child Discipline | Cues Emotional States | |
| Child parent relation | Imitation | |
| Child rearing | Imitative Behavior | |
| Child, Preschool | Mirroring | |
| Childhood Play Behavior | Nurturance | |
| Childrearing Practices | Observation | |
| Dyads | Play and Playthings | |
| Maternal Behavior | Praise | |
| Mother Child Communication | Recreation | |
| Mother child relation | Reinforcement | |
| Mother Child Relations | Reinforcement Schedules | |
| Mother-Child Relations | Reinforcement, Verbal | |
| Parent Training | Slap | |
| Parenting | Smack | |
| Preschool child | Spank | |
| Affect | Time-out or Time out | |
| Behavior disorder | Therapy | |
| Behavior Modification | Training | |
| Behavior Problems | Videorecording | |
| Behavior therapy | Videotape Recording | |
| Compliance |  | |
| Conditioning |  | |
| 1. Child. | |  |
| 2. Child, preschool. | |  |
| 3. Parents. | |  |
| 4. Maternal behavior. | |  |
| 5. Child behavior. | |  |
| 6. Child rearing. | |  |
| 7. Mother-child relations. | |  |
| 8. Mother-child interaction. | |  |
| 9. Father-child relations. | |  |
| 10. Parent-child relations. | |  |
| 11. Parenting. | |  |
| 12. 1 or 2 or 3 or 4 or 5 or 6 or 7 or 8 or 9 or 10 or 11 | |  |
| 13. Imitative. | |  |
| 14. "play*". | |  |
| 15. Reinforcement. | |  |
| 16. Role playing. | |  |
| 17. "video*". | |  |
| 18. Mirroring. | |  |
| 19. Modeling. | |  |
| 20. Praise. | |  |
| 21. "complian*". | |  |
| 22. Time-out. | |  |
| 23. "non-complian*". | |  |
| 24. 13 or 14 or 15 or 16 or 17 or 18 or 19 or 20 or 21 or 22 or 23 | |  |
| 25. "randomized controlled trial".pt. | |  |
| 26. Pragmatic Clinical Trial.pt. | |  |
| 27. Exp Randomized Controlled Trials as Topic/ | |  |
| 28. "Randomized Controlled Trial (topic)"/ | |  |
| 29. Randomized Controlled Trial/ | |  |
| 30. Randomization/ | |  |
| 31. Random Allocation/ | |  |
| 32. Double-Blind Method/ | |  |
| 33. Double Blind Procedure/ | |  |
| 34. Double-Blind Studies/ | |  |
| 35. Single-Blind Method/ | |  |
| 36. Single Blind Procedure/ | |  |
| 37. Single-Blind Studies/ | |  |
| 38. Placebos/ | |  |
| 39. Placebo/ | |  |
| 40. (random* or sham or placebo*). | |  |
| 41. ((singl* or doubl*) adj (blind* or dumm* or mask*)). | |  |
| 42. ((tripl* or trebl*) adj (blind* or dumm* or mask*)). | |  |
