# Supplementary material for: Parenting behaviors that shape child compliance: A multilevel meta-analysis
Source: PLoS One. 2018 Oct 5;13(10):e0204929. doi: 10.1371/journal.pone.0204929 (PMC6173420; doi:10.1371/journal.pone.0204929)
Supplement: S2 Table — (DOCX) [file pone.0204929.s003.docx]

**S2 Table. Additional Search Strategy for Disentangling Trials.**

We identified systematic reviews published from 2008 to 2011 in a previous review^1^ using the search terms below, and updated the search for 2011–2014^2^. (The search terms were also adapted for use in other databases, including CINAHL, *meta*Register of Controlled Trials [*m*RCT] ([*http://www.controlled-trials.com*](http://www.controlled-trials.com)*)*, and the Cochrane Database of Systematic Reviews.)

**Search terms used for PsycINFO (1967 to April 2014), MEDLINE (1948 to April 2014) and EMBASE (1980 to April 2014)**

1. exp "literature review"/ or exp meta analysis/

2. systematic review.mp. [mp=title, abstract, heading word, table of contents, key concepts, original title, tests & measures]

3. 1 or 2

4. exp Parents/ or exp Parent Training/ or exp Mothers/ or exp Childrearing Practices/ or exp Family Relations/ or exp Parenting Skills/

5. exp Parenting/

6. exp Parent Child Relations/

7. exp Conduct Disorder/

8. exp Antisocial Personality Disorder/

9. exp Juvenile Delinquency/

10. exp Child Abuse/

11. exp Child Neglect/

12. exp Mother Child Communication/ or exp Child Discipline/ or exp Child Psychology/ or exp Father Child Relations/ or exp Mother Child Relations/ or exp Parent Child Relations/ or exp Parent Child Communication/ or exp Father Child Communication/

13. Child.mp.

14. Preschool.mp. [mp=title, abstract, heading word, table of contents, key concepts, original title, tests & measures]

15. exp Aggressiveness/ or exp Aggressive Behavior/ or exp Behavior Disorders/

16. exp Antisocial Personality Disorder/ or exp Antisocial Behavior/

17. Offending.mp. [mp=title, abstract, heading word, table of contents, key concepts, original title, tests & measures]

18. (Behavio* and (change or therapy)).mp. [mp=title, abstract, heading word, table of contents, key concepts, original title, tests & measures]

19. 13 or 14

20. 15 or 16 or 17 or 18

21. 19 and 20

22. 4 or 5 or 6 or 7 or 8 or 9 or 10 or 11 or 12 or 21

23. 3 and 22

**Search terms used for PsycINFO (2002 to January 2015), Ovid MEDLINE(R) (1946 to January 2015), Embase (1996 to January 2015)** (Search terms were adapted for use in other databases, including CINAHL and the Cochrane Database of Systematic Reviews.)

1. exp Parents/ or exp Parent Training/ or exp Mothers/ or exp Childrearing Practices/ or exp Family Relations/ or exp Parenting Skills/

2. exp Parenting/

3. exp Parent Child Relations/

4. exp Conduct Disorder/

5. exp Juvenile Delinquency/

6. exp Child Abuse/

7. exp Child Neglect/

8. exp Mother Child Communication/ or exp Child Discipline/ or exp Child Psychology/ or exp Father Child Relations/ or exp Mother Child Relations/ or exp Parent Child Relations/ or exp Parent Child Communication/ or exp Father Child Communication/

9. Child.mp.

10. Preschool.mp. [mp=title, abstract, heading word, table of contents, key concepts, original title, tests & measures]

11. exp Aggressiveness/ or exp Aggressive Behavior/ or exp Behavior Disorders/

12. exp Antisocial Personality Disorder/ or exp Antisocial Behavior/

13. Offending.mp. [mp=title, abstract, heading word, table of contents, key concepts, original title, tests & measures]

14. (Behavio* and (change or therapy)).mp. [mp=title, abstract, heading word, table of contents, key concepts, original title, tests & measures]

15. 9 or 10

16. 11 or 12 or 13 or 14

17. 15 and 16

18. 1 or 2 or 3 or 4 or 5 or 6 or 7 or 8 or 17

19. randomized controlled trial

20. limit 19 to yr="2011 - 2015"

21. limit 20 to ("preschool child (2 to 5 years)" or "child (6 to 12 years)")

22. limit 21 to humans

23. limit 22 to peer reviewed journal

24. limit 23 to treatment & prevention

25. limit 24 to ("core clinical journals (aim)" or communication disorders journals or consumer health journals or foreign journals or health technology assessment journals or nursing journals)

^1^ Gardner F, Montgomery P, Knerr W. Transporting evidence-based parenting programs for child problem behavior (age 3–10) between countries: Systematic review and meta-analysis. *J. Clin. Child Adolesc. Psychol.* 2015:1-14.

^2^ Leijten P, Melendez-Torres GJ, Knerr W, Gardner F. Transported versus homegrown parenting interventions for reducing disruptive child behavior: A multilevel meta-regression study. *JAACAP*. 2016: 55, 233-243
