# Supplementary material for: Parenting behaviors that shape child compliance: A multilevel meta-analysis
Source: PLoS One. 2018 Oct 5;13(10):e0204929. doi: 10.1371/journal.pone.0204929 (PMC6173420; doi:10.1371/journal.pone.0204929)
Supplement: S3 Table — (DOCX) [file pone.0204929.s004.docx]

**S3 Table. Included Studies.**

Acker M, O’Leary S. Inconsistency of mothers' feedback and toddlers' misbehavior and negative affect. J Abnorm Child Psych. 1996;24:703–714.

Adams CD, Kelley ML. Managing sibling aggression: Overcorrection as an alternative to time-out. Behav Ther.1992;23:707-717.

Bean A, Roberts M. The effect of time-out release contingencies on changes in child noncompliance. J Abnorm Child Psych. 1981;9:95–105.

Bernhardt AJ, Forehand R. The effects of labeled and unlabeled praise upon lower and middle class children. J Exp Child Psychol. 1975;19:536–543.

Brock RL, Kochanska G, O'Hara MW, Grekin RS. Life satisfaction moderates the effectiveness of a play-based parenting intervention in low-income mothers and toddlers. J Abnorm Child Psych. 2015;43:1283–1294.

Davies GR, McMahon RJ, Flessati EW, Tiedemann GL. Verbal rationales and modeling as adjuncts to a parenting technique for child compliance. Child Dev. 1984;55:1290–1298.

Eisenstadt TH, Eyberg S, McNeil CB, Newcomb K, Funderburk B. Parent-child interaction therapy with behavior problem children: Relative effectiveness of two stages and overall treatment outcome. J Clin Child Psychol. 1993;22:42–51.

Gardner H, Forehand R, Roberts M. Time-out with children. Effects of an explanation and brief parent training on child and parent behaviors. J Abnorm Child Psych. 1976;4:277–288.

Kochanska G, Kim S, Boldt LJ, Nordling JK. Promoting toddlers' positive social-emotional outcomes in low-income families: A play-based experimental study. J Clin Child Adolesc Psych. 2013;42:700–712.

Leijten P, Thomaes S, Orobio de Castro B, Dishion TJ, Matthys W. What good is labeling what’s good? A field experimental investigation of parental labeled praise and disruptive child behavior. Behav Res Ther. 2016;83:134–141.

O'Dell SL, O'Quin JA, Alford BA, O'Briant AL, Bradlyn AS, Giebenhain JE. Predicting the acquisition of parenting skills via four training methods. Behav Ther. 1982;13:194–208.

Reid MJ, O'Leary S, Wolff L. Effects of maternal distraction and reprimands on toddlers' transgressions and negative affect. J Abnorm Child Psych. 1994;22:237–245.

Reid MJ, Walter AL, O'Leary SG. Treatment of young children's bedtime refusal and nighttime wakings: A comparison of "standard" and graduated ignoring procedures. J Abnorm Child Psych. 1999;27:5–16.

Roberts MW. Praising child compliance: Reinforcement or ritual? J Abnorm Child Psych. 1985;13:611–629. doi: 10.1007/BF00923145

Roberts MW. Enforcing chair timeouts with room timeouts. Behav Modif. 1988;12:353–370.

Roberts MW, Hatzenbuehler LC. Parent treatment of command‐elicited negative verbalizations: A question of persistence. J Clin Child Psychol. 1981;10:107–113.

Scarboro ME, Forehand R. Effects of two types of response-contingent time-out on compliance and oppositional behavior of children. J Exp Child Psychol. 1975;19:252–264.

Wahler RG, Meginnis KL. Strengthening child compliance through positive parenting practices: What works? J Clin Child Psychol. 1997;26:433–440.
