# Supplementary material for: Parenting behaviors that shape child compliance: A multilevel meta-analysis
Source: PLoS One. 2018 Oct 5;13(10):e0204929. doi: 10.1371/journal.pone.0204929 (PMC6173420; doi:10.1371/journal.pone.0204929)
Supplement: S4 Table — (DOCX) [file pone.0204929.s005.docx]

**S4 Table. Excluded Studies and Reasons for Exclusion.**

**Excluded Focused Experimental Studies**

| **Study reference** | **Reason for exclusion** |
| --- | --- |
| Berkovits, M. D., O'Brien, K. A., Carter, C. G., & Eyberg, S. M. (2010). Early identification and intervention for behavior problems in primary care: a comparison of two abbreviated versions of parent-child interaction therapy. *Behavior Therapy, 41*, 375-387. | Clinician (not parent) delivered intervention. |
| Day, D., & Roberts, M. (1983). An analysis of the physical punishment component of a parent training program. *Journal of Abnormal Child Psychology, 11*, 141-152. | Conditions were too similar across conditions for meaningful comparison. |
| Hamilton, S. B., & MacQuiddy, S. L. (1984). Self-administered behavioral parent training: Enhancement of treatment efficacy using a time-out signal seat. *Journal of Clinical Child Psychology, 13*, 61-69. | Conditions were too similar across conditions for meaningful comparison. |
| Hobbs, S. A., & Forehand, R. (1975). Effects of differential release from time out on children's deviant behavior. *Journal of Behavior Therapy and Experimental Psychiatry, 6*, 256-257. | Conditions were too similar across conditions for meaningful comparison. |
| Hobbs, S. A., Forehand, R., & Murray, R. G. (1978). Effects of various durations of timeout on the noncompliant behavior of children. *Behavior Therapy, 9*, 652-656. | Insufficient data provided. |
| Hobbs, S. A., & Forehand, R. (1977). Important parameters in the use of timeout with children: A re-examination. *Journal of Behavior Therapy and Experimental Psychiatry, 8*, 365-370. | Conditions were too similar across conditions for meaningful comparison. |
| Hobbs, S. A., Walle, D. L., & Caldwell, H. S. (1984). Maternal evaluation of social reinforcement and time-out: Effects of brief parent training. *Journal of Consulting and Clinical Psychology, 52*, 135-136. | Conditions were too similar for meaningful comparison, and/or did not compare microtrials, and/or reported insufficient data. |
| Kotler, J. S., & McMahon, R. J. (2004). Compliance and noncompliance in anxious, aggressive, and socially competent children: The impact of the child's game on child and maternal behavior. *Behavior Therapy*, *35*, 495-512. | Tests 1 intervention in 3 different samples, with no control. Thus, not one random sample, but 3 different samples getting the same intervention |
| Lay, K. L., Waters, E., & Park, K. A. (1989). Maternal responsiveness and child compliance: the role of mood as a mediator. *Child Development, 60*, 1405-1411. | Experimenter (rather than parent) delivered the intervention. |
| O'Dell, S. L., Krug, W. W., O'Quin, J. A., & Kasnetz, M. (1980). Media-assisted parent training: A further analysis. *Behavior Therapist*, *3*, 19-21. | Could not locate paper. |
| Powers, S. W., & Roberts, M. W. (1995). Simulation training with parents of oppositional children: Preliminary findings. *Journal of Clinical Child Psychology*, *24*, 89-97. | (in Experiment 2) Conditions were too similar across for meaningful comparison. |
| Roberts, M. W. (1983). The Effects of Warned Versus Unwarned Time-Out Procedures on Child Noncompliance. *Child & Family Behavior Therapy, 4*, 37-53. | Conditions were too similar across conditions for meaningful comparison. |
| Roberts, M. W. (1988). Enforcing Chair Timeouts with Room Timeouts. *Behavior Modification, 12*, 353-370. | Conditions were too similar across conditions for meaningful comparison. |
| Roberts, M. W., & Powers, S. W. (1990). Adjusting chair timeout enforcement procedures for oppositional children. *Behavior Therapy, 21*, 257-271. | Conditions were too similar across conditions for meaningful comparison. |
| Sajwaj, T. E., Pinkston, S., Cordua, G., Jackson, C., Herbert, E. W., Pinkston, E. M., & Hayden, M. L. (1973). Adverse effects of differential parental attention. *Journal of Applied Behavior Analysis, 6*, 15-30. | Not randomized. |
| Ward, S. (2000). Parenting, conduct problems and the development of conscience in young children. *Unpublished thesis*. | Manipulated parenting behavior did not fit with any of the perspectives. |

**Excluded Disentangling trials**

| Webster-Stratton C. (1994). Advancing videotape parent training: a comparison study. *Journal of Consulting and Clinical Psychology, 62,* 583-93. | Tested the effects of different delivery methods to teach parenting behaviors, rather than different parenting behaviors |
| --- | --- |
| Sanders, M. R., Dittman, C. K., Farruggia, S. P., & Keown, L. J. (2014). A comparison of online versus workbook delivery of a self-help positive parenting program. *The Journal of Primary Prevention*, *35*, 125-133. | Tested the effects of different delivery methods to teach parenting behaviors, rather than different parenting behaviors |
| Stanger, C., Ryan, S. R., Fu, H., & Budney, A. J. (2011). Parent training plus contingency management for substance abusing families: A Complier Average Causal Effects (CACE) analysis. *Drug and Alcohol Dependence*, *118*, 119-126. | Tested the effects of different delivery methods to teach parenting behaviors, rather than different parenting behaviors |
| Jones, S., Calam, R., Sanders, M., Diggle, P. J., Dempsey, R., & Sadhnani, V. (2014). A pilot Web based positive parenting intervention to help bipolar parents to improve perceived parenting skills and child outcomes. *Behavioural and Cognitive Psychotherapy, 42,* 283-296. | Tested the effects of different delivery methods to teach parenting behaviors, rather than different parenting behaviors |
| Forehand, R. L., Merchant, M. J., Long, N., & Garai, E. (2010). An examination of Parenting the Strong-Willed Child as bibliotherapy for parents. *Behavior Modification, 34*, 57-76. | Tested the effects of different delivery methods to teach parenting behaviors, rather than different parenting behaviors |
| Salmon, K., Dadds, M. R., Allen, J., & Hawes, D. J. (2009). Can emotional language skills be taught during parent training for conduct problem children? *Child Psychiatry and Human Development, 40*, 485-498. | Tested the additive effects of intervention components other than specific parenting behaviors |
| Salmon, K., Dittman, C., Sanders, M., Burson, R., & Hammington, J. (2014). Does adding an emotion component enhance the Triple P-Positive Parenting Program? *Journal of Family Psychology, 28*, 244-252. | Tested the additive effects of intervention components other than specific parenting behaviors |
| Sanders, M. R., Pidgeon, A. M., Gravestock, F., Connors, M. D., Brown, S., & Young, R. W. (2004). Does parental attributional retraining and anger management enhance the effects of the Triple P-Positive Parenting Program with parents at risk of child maltreatment? *Behavior Therapy, 35,* 513-535. | Tested the additive effects of intervention components other than specific parenting behaviors |
| Ireland, J. L., Sanders, M. R., & Markie-Dodds, C. (2003). The impact of parent training on marital functioning: a comparison of two group versions of the triple p-positive parenting program for parents of children with early-onset conduct problems. *Behavioural and Cognitive Psychotherapy, 31,* 127-142. | Tested the additive effects of intervention components other than specific parenting behaviors |
| Dadds, M. R., & McHugh, T. A. (1992) Social support and treatment outcome in behavioral family therapy for child conduct problems. *Journal of Consulting and Clinical Psychology, 60*, 252-259. | Tested the additive effects of intervention components other than specific parenting behaviors |

**Excluded Single-Subject and Multiple Baseline Studies**

| **Study reference** | **Reason for exclusion** |
| --- | --- |
|  |  |
| Buell, J., Stoddard, P., Harris, F. R., & Baer, D. M. (1968). Collateral social development accompanying reinforcement of outdoor play in a preschool child. *Journal of Applied Behavior Analysis, 1*, 167-173. | Behaviors implemented by experimenters or teachers, rather than parents.  Children with disabilities or disorders other than non-compliance. |
| DeLeon, I. G., Fisher, W. W., Rodriguez‐Catter, V., Maglieri, K., Herman, K., & Marhefka, J. M. (2001). Examination of relative reinforcement effects of stimuli identified through pretreatment and daily brief preference assessments. *Journal of Applied Behavior Analysis, 34*, 463-473. | Behaviors implemented by experimenters or teachers, rather than parents.  Children with disabilities or disorders other than non-compliance. |
| Doleys, D. M., Wells, K. C., Hobbs, S. A., Roberts, M. W., & Cartelli, L. M. (1976). The effects of social punishment on noncompliance: A comparison with timeout and positive practice. *Journal of Applied Behavior Analysis, 9,* 471-482. | Behaviors implemented by experimenters or teachers, rather than parents.  Children with disabilities or disorders other than non-compliance. |
| Ducharme, J. M., Harris, K., Milligan, K., & Pontes, E. (2003). Sequential evaluation of reinforced compliance and graduated request delivery for the treatment of noncompliance in children with developmental disabilities. *Journal of Autism and Developmental Disorders, 33,* 519-526. | Children with disabilities or disorders other than non-compliance. |
| Hanley, G. P., Piazza, C. C., Fisher, W. W., & Maglieri, K. A. (2005). On the effectiveness of and preference for punishment and extinction components of function‐based interventions. *Journal of Applied Behavior Analysis, 38*, 51-65. | Behaviors implemented by experimenters or teachers, rather than parents.  Children with disabilities or disorders other than non-compliance. |
| Kelly, J. A., & Drabman, R. S. (1977). Generalizing response suppression of self-injurious behavior through an overcorrection punishment procedure: A case study. *Behavior Therapy, 8*, 468-472. | Behaviors implemented by experimenters or teachers, rather than parents.  Children with disabilities or disorders other than non-compliance. |
| Plummer, S., Baer, D. M., & LeBlanc, J. M. (1977). Functional considerations in the use of procedural timeout and an effective alternative. *Journal of Applied Behavior Analysis, 10*, 689-705. | Behaviors implemented by experimenters or teachers, rather than parents.  Children with disabilities or disorders other than non-compliance. |
| Russo, D. C., Cataldo, M. F., & Cushing, P. J. (1981). Compliance training and behavioral covariation in the treatment of multiple behavior problems. *Journal of Applied Behavior Analysis, 14*, 209-222. | Behaviors implemented by experimenters or teachers, rather than parents |
| Pinkston, E. M., Reese, N. M., LeBlanc, J. M., & Baer, D. M. (1973). Independent control of a preschool child's aggression and peer interaction by contingent teacher attention. *Journal of Applied Behavior Analysis, 6*, 115-124. | Behaviors implemented by experimenters or teachers, rather than parents |
| Nordquist, V. M., & Wahler, R. G. (1973). Naturalistic treatment of an autistic child. *Journal of Applied Behavior Analysis, 6*, 79-87. | Children with disabilities or disorders other than non-compliance. |
| Budd, K. S., Green, D. R., & Baer, D. M. (1976). An analysis of multiple misplaced parental social contingencies. *Journal of Applied Behavior Analysis, 9*, 459-470. | Children with disabilities or disorders other than non-compliance. |
| Hawkins, R. P., Peterson, R. F., Schweid, E., & Bijou, S. W. (1966). Behavior therapy in the home: Amelioration of problem parent-child relations with the parent in a therapeutic role. *Journal of Experimental Child Psychology, 4*, 99-107. | Effects of packages of parenting behavior |
| Jones, R. N., Sloane, H. N., & Roberts, M. W. (1992). Limitations of “don't” instructional control. *Behavior Therapy, 23*, 131-140 | Effects of packages of parenting behavior |
| Wahler, R. G. (1969). Oppositional children: a quest for parental reinforcement control. *Journal of Applied Behavior Analysis, 2*, 159-170. | Effects of packages of parenting behavior |
| Zeilberger, J., Sampen, S. E., & Sloane, H. N. (1968). Modification of a child's problem behaviors in the home with the mother as therapist. *Journal of Applied Behavior Analysis, 1*, 47-53. | Effects of packages of parenting behavior |
