# Supplementary material for: Parenting behaviors that shape child compliance: A multilevel meta-analysis
Source: PLoS One. 2018 Oct 5;13(10):e0204929. doi: 10.1371/journal.pone.0204929 (PMC6173420; doi:10.1371/journal.pone.0204929)
Supplement: S5 Table — (DOCX) [file pone.0204929.s006.docx]

**S5 Table. Coding Scheme for Classifying Parenting Behaviors.**

| **Parenting behavior** |  | **Criteria** |
| --- | --- | --- |
| Praise |  | **-** Parents are instructed to verbally react to their child.  **-** Parents are instructed to say something nice about the child or his/her behavior (i.e., an expression of approval or admiration). |
| Verbal reprimand |  | **-** Parents are instructed to verbally react to their child.  **-** Parents are instructed to tell the child what s/he did wrong. |
| Time-out |  | **-** Parents are instructed to take the child out of the situation in which noncompliance occurred.  **-** Parents are instructed to place children in a separate part of the room, or another room.  **-** Parents are instructed not to engage in any form of verbal or nonverbal contact with the child for a few minutes. |
| Ignore |  | **-** Parents are instructed not to engage in any form of verbal or nonverbal contact with the child for a few minutes. |
