# Supplementary material for: Parenting behaviors that shape child compliance: A multilevel meta-analysis
Source: PLoS One. 2018 Oct 5;13(10):e0204929. doi: 10.1371/journal.pone.0204929 (PMC6173420; doi:10.1371/journal.pone.0204929)
Supplement: S6 Table — (DOCX) [file pone.0204929.s007.docx]

**S6. Coding Scheme for Classifying Outcome Measures.**

| **Type of outcome** | **Criteria** | **Examples** |
| --- | --- | --- |
| Observed child compliance | Researchers score live or video-taped sessions of the child’s behavior in either structured or unstructured settings, at home or in the lab. | - Frequency counts of noncompliance with parental instructions (e.g., to put certain marbles in certain holes or to clean-up toys). |
| Parent-reported child compliance | Parents reporte on their perceptions of the frequency and/or intensity of incidences of children’s noncompliance at home. | - Eyberg Child Behavior Inventory.  - Home Report Cards. |
